# Generating Giant Membrane Vesicles from Live Cells with Preserved Cellular Properties

**DOI:** 10.34133/2019/6523970

**Published:** 2019-06-17

**Authors:** Qiaoling Liu, Cheng Bi, Jiangling Li, Xuejiao Liu, Ruizi Peng, Cheng Jin, Yang Sun, Yifan Lyu, Hui Liu, Huijing Wang, Can Luo, Weihong Tan

**Affiliations:** ^1^Molecular Science and Biomedicine Laboratory (MBL), State Key Laboratory of Chemo/Bio-Sensing and Chemometrics, College of Biology, College of Chemistry and Chemical Engineering, Aptamer Engineering Center of Hunan Province, Hunan University, Changsha, China; ^2^Institute of Molecular Medicine (IMM), Renji Hospital, Shanghai Jiao Tong University School of Medicine and School of Chemistry and Chemical Engineering, Shanghai Jiao Tong University, Shanghai, China; ^3^Departments of Chemistry, Physiology and Functional Genomics, Molecular Genetics and Microbiology and Pathology and Laboratory Medicine, UF Health Cancer Center, Center for Research at the Bio/Nano Interface, University of Florida, Gainesville, FL, USA

## Abstract

Biomimetic giant membrane vesicles, with size and lipid compositions comparable to cells, have been recognized as an attractive experimental alternative to living systems. Due to the similarity of their membrane structure to that of body cells, cell-derived giant plasma membrane vesicles have been used as a membrane model for studying lipid/protein behavior of plasma membranes. However, further application of biomimetic giant membrane vesicles has been hampered by the side-effects of chemical vesiculants and the utilization of osmotic buffer. We herein develop a facile strategy to derive giant membrane vesicles (GMVs) from mammalian cells in biofriendly medium with high yields. These GMVs preserve membrane properties and adaptability for surface modification and encapsulation of exogenous molecules, which would facilitate their potential biological applications. Moreover, by loading GMVs with therapeutic drugs, GMVs could be employed for drug transport to tumor cells, which represents another step forward in the biomedical application of giant membrane vesicles. This study highlights biocompatible GMVs with biomimicking membrane surface properties and adaptability as an ideal platform for drug delivery strategies with potential clinical applications.

## 1. Introduction

The design and construction of giant vesicles that mimic aspects of cellular structural complexity is an important line of research that has received much recent attention [1]. Biomimetic giant vesicles, with size and lipid composition comparable to cells, have been widely used as membrane models [2], cell models [3, 4], and bioreactors [5, 6]. The conventional strategy of preparing giant vesicles has involved bottom-up engineering of diverse molecular components by self-assembly [7]. While synthetic giant vesicles are relatively easy to prepare, they have largely failed to mimic the complex structures and functions present on the cell surface [8–11]. The preparation of giant vesicles with biomimetic membrane property is still very challenging because of the vast complexity of biological structures [12]. To address these challenges, strategies of producing giant membrane vesicles from live cells were developed.

Cells are capable of generating various vesicles under chemical or physical stimulation [13, 14]. Thus, generating giant cell membrane vesicles from cells presents a unique opportunity for preparing biomimetic giant vesicles. Utilizing chemical vesiculants or osmotic buffer [15–17], giant plasma membrane vesicles (GPMVs) derived from live cells have been used as membrane models for studying lipid/protein behavior of plasma membranes, such as phase separation and functional lipid raft domains [18–22]. While remarkable progress has been made in utilizing GPMVs as a toolbox for research into membrane organization, further biomedical applications of these biomimetic giant membrane vesicles have not been fully exploited. This primarily results from the problems experienced by current approaches of using chemical vesiculants (e.g., formaldehyde and dithiothreitol) that are required for vesicle formation. In addition, due to unwanted chemical reactions induced by chemical vesiculants, possible disruption of membrane structure may occur during the vesicle isolation process, comprising the broadest limitation of GPMVs [23]. Thus, a simple approach for large-scale production of giant cell membrane vesicles with preserved membrane surface properties is desirable. In addition, rational design and construction of cell membrane vesicles, which would provide giant cell membrane vesicles with extra functionalities, is especially necessary to extend their further application. Thus, it is an intriguing goal to develop a new strategy to prepare giant membrane vesicles in biocompatible buffer and endow them with extra functionalities, which would greatly facilitate their potential biological applications.

In this work, we developed a nanomaterial-assisted strategy to generate high-quality giant cell membrane vesicles (GMVs). This facile strategy can produce GMVs from various adherent cells in biofriendly medium (e.g., DMEM, 1640 culture medium or DPBS buffer), thus making GMVs compatible for use with any number of subsequent biological applications without separation. Moreover, these GMVs mimic the natural environment of the membrane surface of the live cells from which they originated and possess the adaptability for further functionalization with nucleic acid molecules. Due to their unique properties, GMVs can effectively transport therapeutic drugs, which represents another step forward in the design and functionality of giant membrane vesicles as potential new approach for therapeutic drug delivery.

## 2. Results

### 2.1. Preparation and Characterization of GMVs

**Figure 1:**
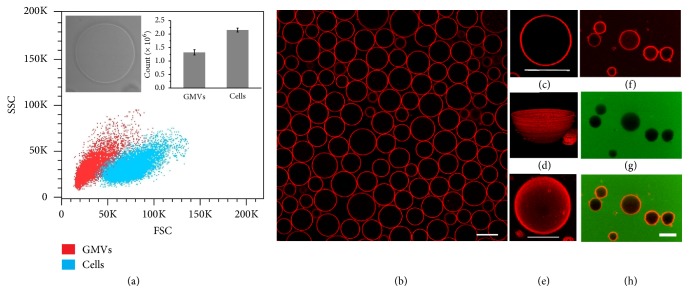
*Flow cytometry analysis and confocal images of GMVs derived from HeLa cells*. (a) Two-dimensional (2D) dot plots of side light scattered area (SSC-A) versus forward light scattered area (FSC-A) from flow cytometry exhibit two distinct populations for HeLa cells (blue dots) and GMVs (red dots). The inset image is the bright field image of a single GMV. (b) Confocal images of GMVs stained with FM 4-64 dye. The confocal image (c), 3D image (d), and Z-stack image (e) of a single vesicle all exhibit spherical structure with good symmetry. (f)-(h) Images of GMVs incubated with fluorescein demonstrate good integrity. Scale bar is 20 *μ*m for (b) and 10 *μ*m for (c)-(h).

Inspired by the observations of plasma membrane vesiculation during light irradiation in our previous report [24], a nanomaterial-assisted strategy was developed to produce high-quality, micrometer-sized GMVs. Typically, HeLa cells were first incubated with carboxylfullerenes then washed and irradiated with white light to induce formation of cell blebs. After overnight incubation, the blebs detached from cells spontaneously and dispersed in supernatant with 5-22 *μ*m size range (supporting information (SI), [Supplementary-material supplementary-material-1]). The supernatant containing GMVs was collected and analyzed by flow cytometry. As shown in Figure 1(a), two-dimensional (2D) dot plots of forward light scattered area (FSC-A) versus side light scattered area (SSC-A) from flow cytometry exhibit two distinct populations for HeLa cells (blue dot) or GMVs derived from HeLa cells (red dot). This facile strategy could produce considerable GMVs from cells. As many as 1.32 × 10^6^ GMVs were prepared from 2.15 × 10^6^ HeLa cells, as determined by flow cytometry analysis (Figure 1(a)).

Owing to their large size, GMVs can be observed under a microscope. However, the GMVs are highly transparent and thus difficult to distinguish from the surrounding background under bright field illumination (Figure 1(a), inset). Therefore, to enable easy monitoring, we stained the GMVs with lipophilic styryl fluorescent dye FM 4-64, a common cell membrane dye, which becomes intensely fluorescent after membrane insertion, but nonfluorescent in aqueous media [25]. As shown in Figure 1(b), high-quality GMVs with little cell debris can be obtained in the supernatant of the culture medium. Strong and homogeneous fluorescence indicated the well-preserved phospholipid bilayer of GMVs [26]. High magnification confocal images of a single GMV showed the spherical structure (Figure 1(c)), as well as three-dimensional (Figure 1(d)) and Z-stack (Figure 1(e)) images. Upon addition of fluorescein isothiocyanate (FITC) dye, the absence of significant change of both inner space and membrane of GMVs, as determined by fluorescence intensity from the inner space of GMVs (Figures 1(f)–1(h)), indicated that the integrity of the GMV membrane was intact, thus preventing the entry of FITC dye. The constituents of GMVs were further analyzed utilizing agarose gel electrophoresis, SDS-PAGE, Western blotting, and mass spectrometry. Results showed that GMVs contained a panel of natural biomolecules, such as Na^+^/K^+^-ATPase and a few nucleic acid pieces (SI, [Supplementary-material supplementary-material-1], [Supplementary-material supplementary-material-1]).

Utilizing this strategy, GMVs can be generated in various biocompatible media (e.g., DMEM, 1640 culture medium) or DPBS buffer, making them compatible for use with any number of subsequent biological applications. More importantly, this strategy also works nicely for various adherent cell types, either cancerous cells or noncancerous cells. We have tested several cancerous cells, such as HepG2 and MCF-7 cells, as well as noncancerous cells, such as HEK 293 and DC2.4 cells. All these cells can be used to produce high-quality GMVs (SI, [Supplementary-material supplementary-material-1]). Thus, instead of chemical vesiculants or osmotic salt buffer, our method differs in that the nanomaterials work as the “vesiculant reagent”. This strategy avoids the unwanted chemical reactions on membrane surfaces and can generate vesicles in biocompatible buffer efficiently.

### 2.2. GMVs Preserve Cellular Surface Properties

**Figure 2:**
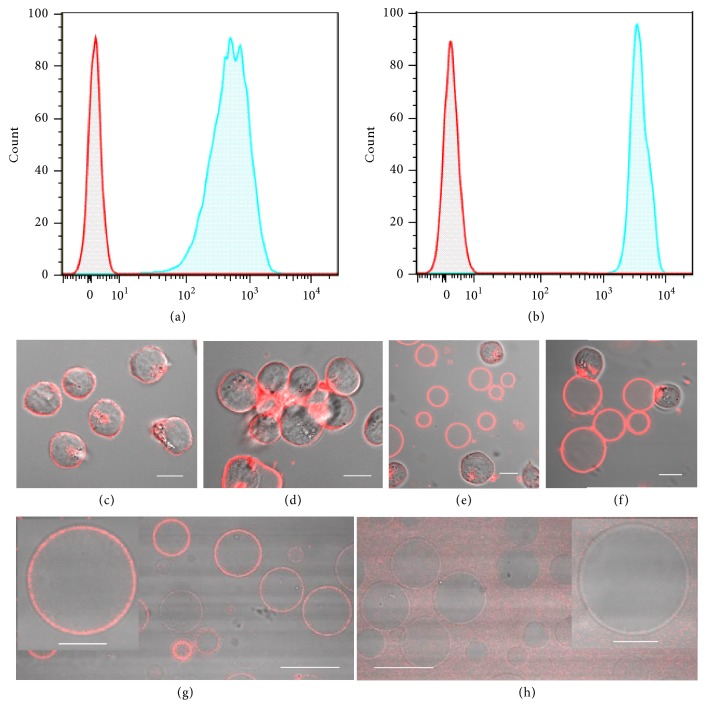
*GMVs exhibit well-preserved membrane properties*. Flow cytometry analysis of (a) GMVs and (b) HeLa cells incubated with fluorescein-labeled WGA. Confocal images of CCRF-CEM cells and GMVs demonstrate the similar response to WGA-induced aggregation. Overlay images of bright field and fluorescent images of CCRF-CEM cells (c) before and (d) after incubation with WGA. Mixture of CCRF-CEM cells and GMVs (e) before and (f) after incubation with WGA. The overlay images of GMVs incubated with PE-labeled Mouse anti-Human CD71 antibody (g) or Mouse IgG 2a (h) demonstrate the good recognition capability of CD71 on GMVs. About 10000 events were counted for each sample. Scale bar is 30 *μ*m for (g), (h) and 10 *μ*m for (c)-(f) and the inset figures in (g) and (h).

To clarify whether GMVs preserve cell surface properties similar to those of live cells, we tested the specific binding capability of glycoconjugates and membrane proteins on the surfaces of GMVs. Herein, wheat germ agglutinin (WGA), a glycan-binding protein, was selected because of its specific recognition by sialic acid and N-acetyl glucosamine moieties on the cell surface [27]. As shown in Figure 2(a), the strong binding between fluorescein-labeled WGA and GMVs demonstrated the preservation of glycans on the surface of GMVs that originated from HeLa cells, since HeLa cells also exhibit strong binding ability to fluorescein-labeled WGA (Figure 2(b)). Moreover, GMVs show WGA-induced cellular aggregation similar to that of living cells. The transformation of well-dispersed morphology into obvious aggregation occurred after incubating GMVs with living CCRF-CEM cells in the presence of WGA (Figure 2(f)), indicating preserved biological function of glycans on the surfaces of GMVs. Thus, we speculated that GMVs possess good recognition capability of surface glycoconjugates, thus presenting a response to WGA-induced interaction similar to that of living cells. In addition to glycoconjugates, the specific binding capability of membrane protein on the surfaces of GMVs was also tested. Herein, transferrin receptor CD71, a cell surface protein which is implicated in the carcinogenesis of various types of tumors, was selected [28, 29]. GMVs incubated with either PE-labeled Mouse anti-Hunan CD71 antibody or Mouse IgG 2a were observed by confocal microscopy. The confocal images demonstrated that GMVs selectively bind to anti-Human CD 71 (Figure 2(g)) instead of IgG 2a (Figure 2(h)), indicating the preserved recognition capability of CD71 on the membrane surfaces of GMVs. Thus, the preserved cell surface properties of GMVs verified our feasible strategy of producing high-quality GMVs and could facilitate their further biological applications.

### 2.3. GMVs Exhibit the Adaptability for Modification with Exogenous Molecules

**Figure 3:**
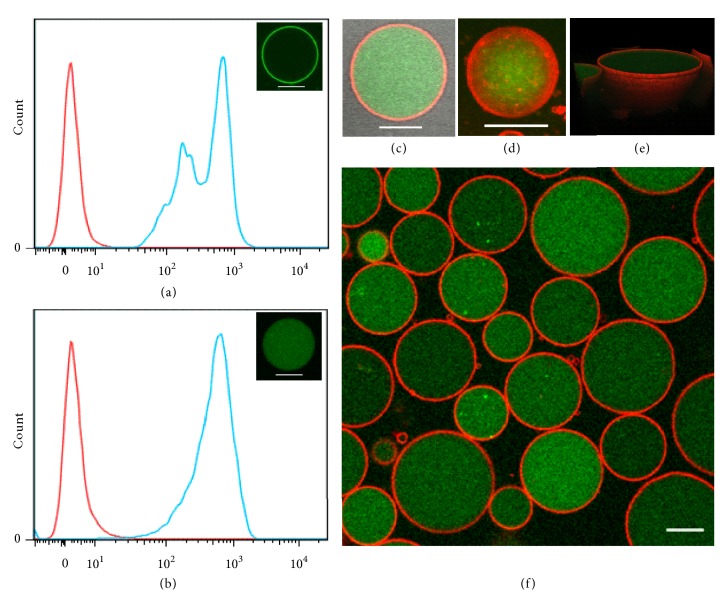
*Modification of GMVs with fluorescein-labeled ssDNA.* Flow cytometry analysis and confocal image of GMVs incubated with (a) fluorescein-labeled PolyT10-lipid or (b) fluorescein-labeled ssDNA (L77). (c) GMVs encapsulating fluorescein-labeled L77 were stained with FM 4-64 dye. The Z-stack image (d) and 3D image (e) of a single giant vesicle are also shown. The overlay images of green fluorescence and red fluorescence demonstrate that fluorescein-labeled L77 was mainly located inside GMVs (f). Scale bar is 10 *μ*m.

As a part of the cell membrane, GMVs have membrane composition similar to that of the native plasma membrane, thus providing an ideal platform for surface modification of GMVs. Herein, diacyllipid-conjugated oligonucleotides were used, since the diacyllipid could efficiently insert into the cell membrane based on hydrophobic interactions between the lipophilic tail and cellular phospholipid bilayer [30]. As shown in Figure 3(a), obvious shift of fluorescence intensity can be observed for GMVs incubated with fluorescein-labeled diacyllipid-PolyT_10_ conjugates for 0.5 h at 37°C. Confocal imaging confirmed the surface location of diacyllipid-PolyT_10_ conjugate (Figure 3(a) insert). Aside from the diacyllipid-PolyT_10_ conjugate, cholesterol-conjugated PolyT_10_ was also used to modify the surfaces of GMVs, and, as expected, it efficiently bound on the surface of GMVs, indicating the feasible manipulation of GMVs with exogenous lipid compounds ([Supplementary-material supplementary-material-1]).

Moreover, GMVs could concentrate the dispersive single-stranded DNA (ssDNA, termed L77). As shown in Figure 3(b), fluorescein-labeled L77 could enter into GMVs after overnight incubation at 37°C. The high magnification image (Figure 3(c)), Z-stack image (Figure 3(d)), and three-dimensional image (Figure 3(e)) shows the spherical structure of GMVs containing the fluorescein-labeled ssDNA inside and FM 4-64-labeled membrane outside (Figure 3(f)). After encapsulation, the fluorescein-labeled L77 was retained in GMVs, and fluorescence could still be detected after several days of storage at 37°C (SI, [Supplementary-material supplementary-material-1]). The mechanism of the sequestration of L77 into GMVs is an ongoing subject of interest in our lab. However, we ascribe this new finding to the well-preserved biological activity of GMVs. Taken together, these data suggest that GMVs possess good adaptability for surface modification and encapsulation of exogenous molecules.

### 2.4. GMVs as Potential Carriers for Drug Transport

**Figure 4:**
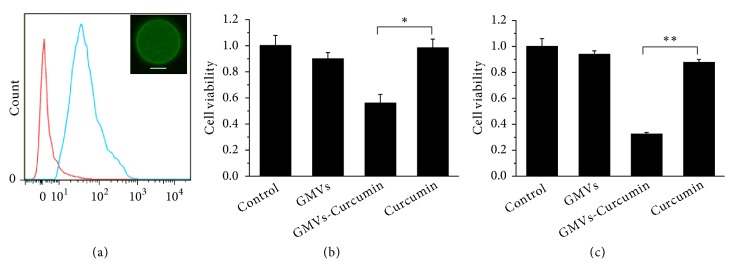
*GMVs as packaging of therapeutic drugs for drug delivery*. Flow cytometry of GMVs loaded with curcumin (a). Cell viability of HeLa cells incubated with GMVs loaded with curcumin (0.5 *μ*g mL^−1^) for 48 h (b). Cell viability of CCRF-CEM cells incubated with GMVs loaded with curcumin (4 *μ*g mL^−1^) for 24 h (c). About 10000 events were counted for each sample. Scale bar is 10 *μ*m. The statistical significance of the observed differences was analyzed by t tests. Statistical significance was set at p < 0.05 (*∗*P < 0.05, *∗∗*P < 0.01).

Considering the membrane vesicle's capability as drug carrier [31, 32], we expected that the GMVs could be used as a versatile carrier for efficient therapeutic drug delivery. Curcumin has been well documented as a potent anti-inflammatory, antineoplastic, and chemopreventive reagent [33, 34]. However, the bioavailability and clinical efficacy of curcumin are limited by its poor solubility [35]. Herein, we tested the feasibility of GMVs as drug carriers to enhance the therapeutic effect of curcumin. Given the fluorescent nature of curcumin, we employed confocal microscopy and flow cytometry to determine whether curcumin could be packaged into GMVs. As shown in Figure 4(a), flow cytometry analysis of GMVs treated with curcumin showed strong fluorescence intensity, indicating that curcumin can be efficiently loaded by GMVs. The efficient loading of curcumin in GMVs indicates potential applications in drug delivery systems (SI, [Supplementary-material supplementary-material-1]).

To clarify the efficiency of GMV-mediated drug delivery, GMVs derived from HeLa cells were loaded with curcumin and incubated with HeLa cells in the presence of fetal bovine serum (FBS, 10 %). Cell viability was tested after incubation for 48 h at 37°C. In the GMV-drug group, cells showed obvious cell death. In contrast, in the free drug group, the cell death was much weaker (Figure 4(b)). As a control, GMVs generated from HeLa cells had no discernible cytotoxic effect. Moreover, GMVs could enhance the therapeutic effect of curcumin for suspension tumor cells. As shown in Figure 4(c), while 4 *μ*g mL^−1^ curcumin killed only about 12 % CCRF-CEM tumor cells, GMVs loaded with the curcumin induced around 68 % tumor cell death, suggesting that drug encapsulation in GMVs may have a higher tumor-killing efficacy than conventional therapeutic drugs.

## 3. Discussion

The unique advantage of giant membrane vesicles compared with synthetic giant vesicles is the true composition of the biological plasma membrane. Thus, the preservation of membrane surface properties is the key issue in the preparation of giant membrane vesicles. We herein developed a nanomaterial-assisted strategy to prepare high-quality giant membrane vesicles. This strategy avoids the use of chemical vesiculants (formaldehyde and dithiothreitol) which would cause unwanted chemical reactions during the preparation. More importantly, GMVs can be produced in biofriendly buffers such as DMEM or 1640 culture medium, making them compatible for use with any number of subsequent biological applications without isolation procedures. Based on the preserved biomimetic membrane surface, a feasible functionalization strategy which utilizes the insertion of a lipid tail into the lipid bilayer of GMVs and encapsulation of ssDNA was achieved to provide GMVs with extra functionalities and extend their further application.

This reliable strategy can produce a large number of GMVs from live cells, thus indicating the promise of biomedical applications of GMVs. Due to membrane vesicles' natural role in transporting bioactive drugs, we tested the feasibility of GMVs as drug carriers and found that use of GMVs for therapeutic drug delivery could efficiently increase the toxicity of drugs in tumor cells. Whether and how GMVs synergize with the killing of tumor cells is currently under study. However, more important for the purposes of this paper is that a facile strategy was developed to prepare GMVs with biomimetic membrane surfaces and that GMVs show promise as simple effective drug carriers for therapeutic interventions in dealing with tumor cells. Therefore, GMVs may well represent a new and efficient approach to deliver therapeutic agents.

In summary, we have developed a facile strategy for generating cell membrane-based giant vesicles capable of (1) behaving at the cell surface in a manner similar to that of the source cells from which they originated and (2) adapting to encapsulation and surface modification, thus adding full functionality to biomimetic membrane properties. This strategy is accessible to most adherent cell types and could produce GMVs with high yield and purity. By virtue of the biomimetic membrane properties, we provided direct evidence that use of GMVs for packaging of therapeutic drugs can contribute to cancer therapy as drug carrier and can efficiently increase the toxicity of drugs in tumor cells. This study may potentially revolutionize the field with regard to the investigation and application of cell-derived giant membrane vesicles and meanwhile open new avenues for generating multifunctional drug carriers.

## 4. Materials and Methods

### 4.1. Cell Culture and Preparation of ssDNA

HeLa, HepG2, MCF-7, HEK-293, DC2.4, and CCRF-CEM cells were all cultured with either Dulbecco's Modified Eagle's Medium (DMEM) or 1640 culture medium (Life Technologies, USA) supplemented with 10 % fetal bovine serum (Hyclone Company, South Logan, UT), penicillin (100 *μ*g mL^−1^), and streptomycin (100 *μ*g mL^−1^; Gibco, Grand Island, NY, USA) in 5 % CO_2_ at 37°C in a humidified incubator.

FITC-labeled ssDNA was purchased from Sangon Biotechnology (Shanghai, China). The sequence of ssDNA (L77) was 5' FITC-TGT GTG TGT GTG TGT GTG TGT GTG TGT GTG TGT GTG TGT GTG TGT GTG TGTGTG TGT GTG TGT GTG TGT GTG TGT GT 3', and the FITC-labeled cholesterol-Poly T_10_ (5' TTT TTT TTTT 3') conjugate was also purchased from Sangon Biotechnology (Shanghai, China).

### 4.2. Preparation and Characterization of GMVs

Cells were seeded into 60-mm culture plates at a density of 2×10^5^ cells and grown for 24 h. For the preparation of GMVs, cells were incubated with 50 *μ*L carboxylfullerenes (0.5 mg mL^−1^) for 3 h at 37°C in 1640 or DMEM culture medium and then washed twice with PBS. Blebbing was initiated by white light irradiation for 30 min under a metal halide light source, and then the suspended GMVs were collected for use. To identify GMVs from the surrounding background, 0.5 mL GMVs were stained with 1 *μ*L FM 4-64 (1 mg mL^−1^, Life Technologies, USA) for 5 min at room temperature and observed by confocal microscopy. Images of GMVs were collected, and their size distribution was calculated by Image J software. Statistical analysis using Origin 8.1 software determined the size distribution of GMVs by analysis of more than 500 GMVs.

To verify the yields and membrane integrity of GMVs, a flow cytometry-based method was used to count the number of GMVs. After light irradiation, GMVs suspended in culture medium were collected for flow cytometric analysis. Meanwhile, cells without light irradiation were collected and the number of cells was counted by flow cytometry. To confirm the integrity of GMVs, 1 *μ*L FITC dye (1 mg mL^−1^) was added into GMVs solution for several minutes and then FM 4-64 dye was added into the solution before the observation under confocal microscope at room temperature.

For the analysis of protein and nucleic acid retained in GMVs, gel electrophoresis was used. Briefly, 5 mL of GMV solution was filtered through a 200-nm polycarbonate membrane and concentrated by centrifugation in a centrifugal filter (10 KD, Millipore). DNA analysis of GMVs was performed by electrophoresis on a 3 % (w/v) agarose gel, followed by visualization by staining with ethidium bromide. The genomic DNA of HeLa cells was extracted and used as the control. For protein characterization by SDS-PAGE, the solution of GMVs derived from HeLa cells was concentrated as described above. A sample was prepared at a final protein concentration of 1 mg mL^−1^ in loading buffer (BioTime, China), as measured by BCA assay (Pierce). Samples were heated to 100°C for 10 min, and 20 *μ*L of sample was loaded into each well of an SDS-PAGE 10 % Bis-Tris 10-well minigel in running buffer in an electrophoresis system (Bio-RAD, USA) based on the manufacturer's instructions. Protein staining was accomplished using Colloidal Blue and destained in water overnight before imaging.

For western blot analysis, GMVs derived from HeLa cells were used and treated as described above. Protein concentrations were determined by a BCA protein assay kit (PIERCE, Rockford, IL, USA). Standard Western blotting was done with a rabbit antibody against Na^+^/K^+^-ATPase (3010S, Cell Signaling Technology), *α*-tubulin (ab15246-500, abcam) or RCC-1 (3784-1, Epitomics, Inc.), along with anti-rabbit IgG (sc-2004, Santa Cruz). Whole cell lysates were prepared from HeLa cells and used as the control.

For mass spectrometric analysis, proteins in individual bands manually excised from the gels after SDS-PAGE were subjected to trypsin digestion and analyzed by liquid chromatography-tandem mass spectrometry (LC-MS/MS) on an LTQ-Orbitrap Velos (Thermo Fisher Scientific, San Jose, CA). Proteome Discoverer (PD), version 1.4.1.14 (Thermo-Scientific), was used to perform the database search against the respective Swiss-Prot protein database (June 2012 version) for these raw data files. The search engines SEQUEST-HT, Mascot (version 2.4.0), and MS Amanda (version 1.4.4.2822) were implemented in PD as previously described [36].

### 4.3. Characterization of Surface Properties of GMVs

To test the recognition capability of glycoconjugates on the surfaces of GMVs, 2 *μ*L fluorescein-labeled WGA was incubated with GMVs at 37°C for 1 h, and then the sample was observed under confocal microscopy directly. For WGA-induced cell aggregation, 10 *μ*L WGA (1 mg mL^−1^) was added to CCRF-CEM cells with or without GMVs at 37°C for 2 h. Subsequently, samples were stained with FM 4-64 as described above and observed by confocal microscopy. To test the recognition capability of transferrin receptor CD71 on the membrane surfaces of GMVs, PE Mouse Anti-Human CD71 (BD Pharmingen) and PE Mouse IgG 2a *κ* Isotype (BD Pharmingen) were incubated with GMVs separately, and then the sample was observed under confocal microscopy directly. For the modification of GMVs with fluorescent dye-labeled ssDNA, 0.5 mL GMVs solution was incubated with 20 *μ*L fluorescent dye-labeled ssDNA (10 *μ*M) at 37°C for 12 h or 10 *μ*L FAM-labeled DNA-lipid conjugates (10 *μ*M) at 37°C for 1 h. Samples were imaged under confocal microscopy or analyzed by flow cytometry after incubation.

### 4.4. Flow Cytometric Analysis

To test the recognition capability of glycoconjugates on the surfaces of GMVs derived from HeLa cells, 2 *μ*L fluorescein-labeled WGA (1 mg mL^−1^) was incubated with GMVs at 37°C for 1 h and then the sample was analyzed by flow cytometry. To verify the entrapment of fluorescent dye-labeled ssDNA or curcumin in GMVs, 0.5 mL GMVs was incubated with either curcumin (10 *μ*g mL^−1^) for 1 h or 20 *μ*L fluorescein-labeled ssDNA (10 *μ*M) for 12 h at 37°C and the samples were analyzed by flow cytometry (BD FACS Verse™ flow cytometer). About 10000 events were counted for each of the samples.

### 4.5. Confocal Microscopy Imaging of GMVs and Cells

For confocal imaging of GMVs, 0.5 mL of sample treated as above was dropped onto a 35-mm glass bottom dish and imaged by a FV1000-IX81 Confocal Laser Scanning Microscope (Olympus, Japan) or a LSM 880 confocal microscope (Zeiss, Germany). For cell imaging, cells were treated in a 35-mm glass bottom dish and imaged using a FV1000-X81 confocal microscope (Olympus, Japan). The images were analyzed by FV10-ASW, Version 3.1.

### 4.6. Cell Viability Assay

All experiments were conducted in 10 % FBS-containing 1640 culture medium. GMVs derived from HeLa cells were obtained. Cells were incubated with GMVs loaded with curcumin for 24 h or 48 h, and cells without incubation of GMVs or incubation with free drugs were used as control. After incubation, cell viability was evaluated using a WST-8 assay with Cell Counting Kit-8 (CCK-8; DOJINDO, Kumamoto, Japan). The absorbance value at 450 nm was read with a 96-well plate reader (iMark™ microplate absorbance reader, Bio-Rad) to determine viability (cell viability = (OD_tre_-OD_medium_)/(OD_con_-OD_medium_), where OD_tre_ is the absorbance value at 450 nm of treated cells, OD_con_ is that of control cells, and OD_medium_ is that of the culture medium.

### 4.7. Statistical Analysis

All the experiments were performed in triplicate. Results are expressed as means ± standard deviation of the mean value (SD). The statistical significance of the observed differences was analyzed by t tests. Statistical significance was set at p < 0.05 (*∗*P < 0.05, *∗∗*P < 0.01).

## Supplementary Materials

Supplementary Materials
* Figure S1. Size distribution of GMVs:* the newly produced GMVs show microsized diameters in the size distribution range of 5-22 *μ*m.* Figure S2. Analysis of DNA and protein retained in GMVs:* agarose gel electrophoresis (a) indicates the negligible presence of genomic DNA on GMVs (lane 1, marker; lane 2, genomic DNA extracted from cells; lane 3, DNA on GMVs). SDS-PAGE electrophoresis indicates an abundance of proteins on GMVs (b, lane 1, marker; lane 2, proteins on giant vesicles). Western blotting analysis indicates the presence of membrane-specific marker Na^+^/K^+^-ATPase on GMVs (c, lane 2). The sporadic presence of both *α*-tubulin (a cytosolic marker) and RCC1 (regulator of chromosome condensation 1, a nuclear marker) was detected on the GMVs (c, lane 2). As a control, total proteins extracted from cells show the existence of Na^+^/K^+^-ATPase, *α*-tubulin, and RCC1 (c, lane 1).* Figure S3. Confocal images of GMVs stained with FM 4-64 dye*: GMVs can be obtained from HEK-293 cells (a) (b); DC 2.4 cells (c) (d); MCF-7 cells (e) (f); and HepG2 cells (g)(h). The scale bar is 20 *μ*m for (a), (c), (e), and (g) and 10 *μ*m for (b), (d), (f), and (h).* Figure S4. Confocal microscopy and flow cytometry analysis of GMVs incubated with Alexa 488 labeled ssDNA-cholesterol:* flow cytometry of GMVs modified with Alexa 488-labeled ssDNA-cholesterol (a). Confocal image of GMVs incubated with Alexa 488-labeled ssDNA-cholesterol for 1 h (b). About 10000 events were counted for the sample. Scale bar is 20 *μ*m.* Figure S5. Confocal images of GMVs encapsulating fluorescein-labeled ssDNA:* GMVs incubated with fluorescein-labeled ssDNA for 2 days ((a)-(d)) and 4 days ((e)-(h)) at 37°C. Scale bar is 10 *μ*m for (a)-(c), (e)-(g) and 20 *μ*m for (d) and (h).* Figure S6. Fluorescence intensity of GMVs incubated with 10 μg mL*^*-1*^* curcumin:* the packaging of curcumin in GMVs was characterized by calculating the fluorescence intensity inside (GMVs) and outside (background) of GMVs. More than 80 GMVs were evaluated. Scale bar is 10 *μ*m.* Table S1. List of proteins retained in GMVs, as identified by mass spectrometry:* more than one thousand proteins were identified and the list below contains the 262 membrane proteins among the total.
