# Supplementary material for: Generating Giant Membrane Vesicles from Live Cells with Preserved Cellular Properties
Source: Research (Wash D C). 2019 Jun 17;2019:6523970. doi: 10.34133/2019/6523970 (PMC6750080; doi:10.34133/2019/6523970)
Supplement: Supplementary Materials — Figure S1. Size distribution of GMVs: the newly produced GMVs show microsized diameters in the size distribution range of 5-22 μm. Figure S2. Analysis of DNA and protein retained in GMVs: agarose gel electrophoresis (a) indicates the negligible presence of genomic DNA on GMVs (lane 1, marker; lane 2, genomic DNA extracted from cells; lane 3, DNA on GMVs). SDS-PAGE electrophoresis indicates an abundance of proteins on GMVs (b, lane 1, marker; lane 2, proteins on giant vesicles). Western blotting analysis indicates the presence of membrane-specific marker Na+/K+-ATPase on GMVs (c, lane 2). The sporadic presence of both α-tubulin (a cytosolic marker) and RCC1 (regulator of chromosome condensation 1, a nuclear marker) was detected on the GMVs (c, lane 2). As a control, total proteins extracted from cells show the existence of Na+/K+-ATPase, α-tubulin, and RCC1 (c, lane 1). Figure S3. Confocal images of GMVs stained with FM 4-64 dye: GMVs can be obtained from HEK-293 cells (a) (b); DC 2.4 cells (c) (d); MCF-7 cells (e) (f); and HepG2 cells (g)(h). The scale bar is 20 μm for (a), (c), (e), and (g) and 10 μm for (b), (d), (f), and (h). Figure S4. Confocal microscopy and flow cytometry analysis of GMVs incubated with Alexa 488 labeled ssDNA-cholesterol: flow cytometry of GMVs modified with Alexa 488-labeled ssDNA-cholesterol (a). Confocal image of GMVs incubated with Alexa 488-labeled ssDNA-cholesterol for 1 h (b). About 10000 events were counted for the sample. Scale bar is 20 μm. Figure S5. Confocal images of GMVs encapsulating fluorescein-labeled ssDNA: GMVs incubated with fluorescein-labeled ssDNA for 2 days ((a)-(d)) and 4 days ((e)-(h)) at 37°C. Scale bar is 10 μm for (a)-(c), (e)-(g) and 20 μm for (d) and (h). Figure S6. Fluorescence intensity of GMVs incubated with 10 μg mL-1 curcumin: the packaging of curcumin in GMVs was characterized by calculating the fluorescence intensity inside (GMVs) and outside (background) of GMVs. More than 80 GMVs were evaluated. Scale b [file 6523970.f1.docx]

**Supporting Information**

Generating Giant Membrane Vesicles from Live Cells with Preserved Cellular Properties

Bio-mimicking Giant Vesicles Derived from Cells

Qiaoling Liu^1^*, Cheng Bi^1^, Jiangling Li^1^, Xuejiao Liu^1^, Ruizi Peng^1^, Cheng Jin^1^, Yang Sun^1,2^, Yifan Lyu^1,2^, Hui Liu^1^, Huijing Wang^1^, Can Luo^1^, Weihong Tan^1,2,3^*

^1^ Molecular Science and Biomedicine Laboratory (MBL), State Key Laboratory of Chemo/Bio-Sensing and Chemometrics, College of Biology, College of Chemistry and Chemical Engineering, Aptamer Engineering Center of Hunan Province, Hunan University, Changsha, China

^2^ Institute of Molecular Medicine (IMM), Renji Hospital, Shanghai Jiao Tong University School of Medicine, and School of Chemistry and Chemical Engineering, Shanghai Jiao Tong University, Shanghai, (China)

^3^ Departments of Chemistry, Physiology and Functional Genomics, Molecular Genetics and Microbiology and Pathology and Laboratory Medicine, UF Health Cancer Center, Center for Research at the Bio/Nano Interface, University of Florida, Gainesville, FL, USA

^⋆^ Correspondence should be addressed to Weihong Tan; [tan@chem.ufl.edu](mailto:tan@chem.ufl.edu); Qiaoling Liu; qlliu@iccas.ac.cn

**Supporting Figures (Figure S1-S6) and Table (Table S1)**

**Figure S1.** Size distribution of GMVs. The newly produced GMVs show microsized diameters in the size distribution range of 5-22 μm.


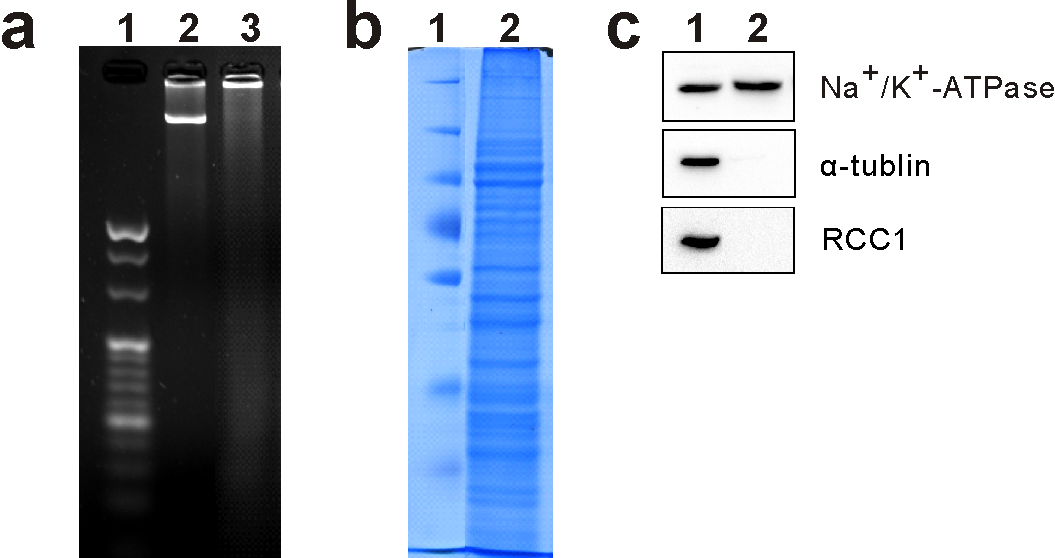


**Figure S2.** Analysis of DNA and protein retained in GMVs. Agarose gel electrophoresis (a) indicates the negligible presence of genomic DNA on GMVs (lane 1, marker; lane 2, genomic DNA extracted from cells; lane 3, DNA on GMVs). SDS-PAGE electrophoresis indicates an abundance of proteins on GMVs (b, lane 1, marker; lane 2, proteins on giant vesicles). Western blotting analysis indicates the presence of membrane-specific marker Na^+^/K^+^-ATPase on GMVs (c, lane 2).The sporadic presence of both α-tublin (a cytosolic marker) and RCC1 (Regulator of chromosome condensation 1, a nuclear marker) was detected on the GMVs (c, lane 2). As a control, total proteins extracted from cells show the existence of Na^+^/K^+^-ATPase, α-tublin and RCC1 (c, lane 1).


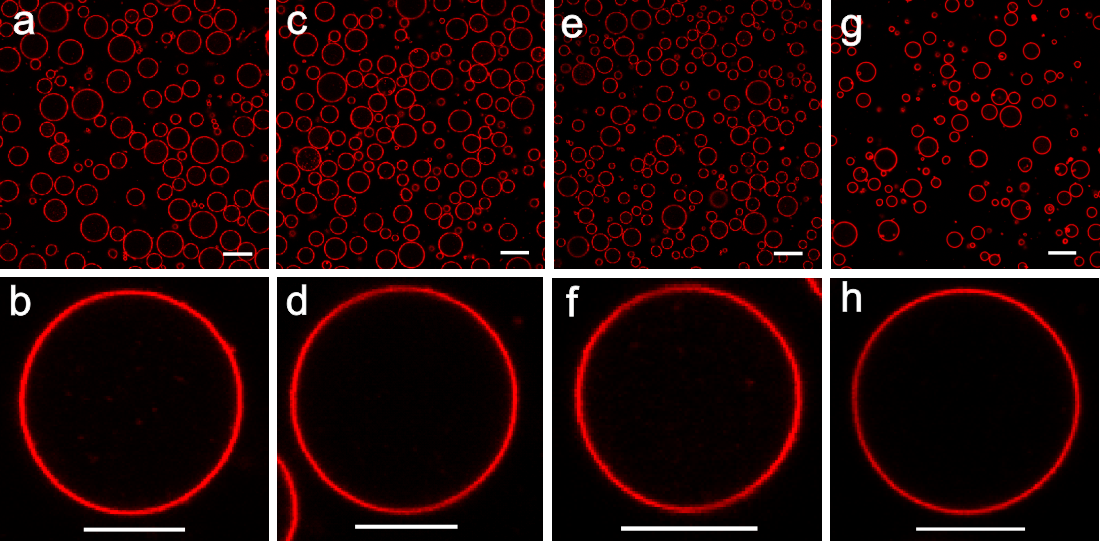


**Figure S3.** Confocal images of GMVs stained with FM 4-64 dye. GMVs can be obtained from HEK-293 cells (a) (b); DC 2.4 cells (c) (d); MCF-7 cells (e) (f); HepG2 cells (g)(h). The scale bar is 20 µm for (a), (c), (e), and (g) and 10 µm for (b), (d), (f), and (h).

**
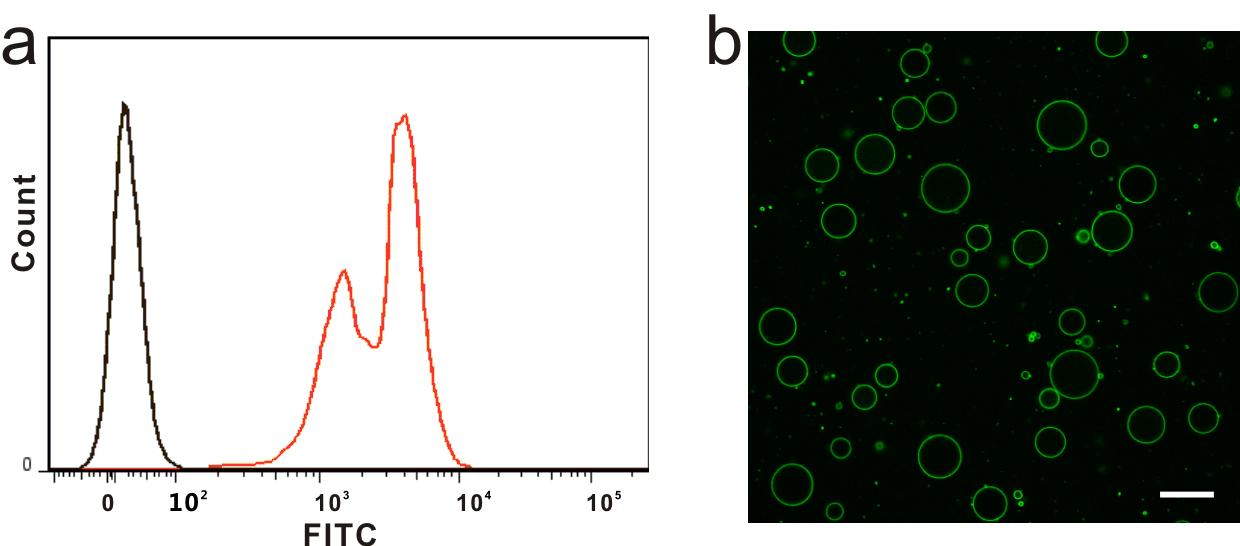
**

**Figure S4.** Confocal and flow cytometry analysis of GMVs incubated with Alexa 488 labeled ssDNA-cholesterol. Flow cytometry of GMVs modified with Alexa 488-labeled ssDNA-cholesterol (a). Confocal image of GMVs incubated with Alexa 488-labeled ssDNA-cholesterol for 1 h (b). About 10000 events were counted for the sample. Scale bar is 20 µm.

**
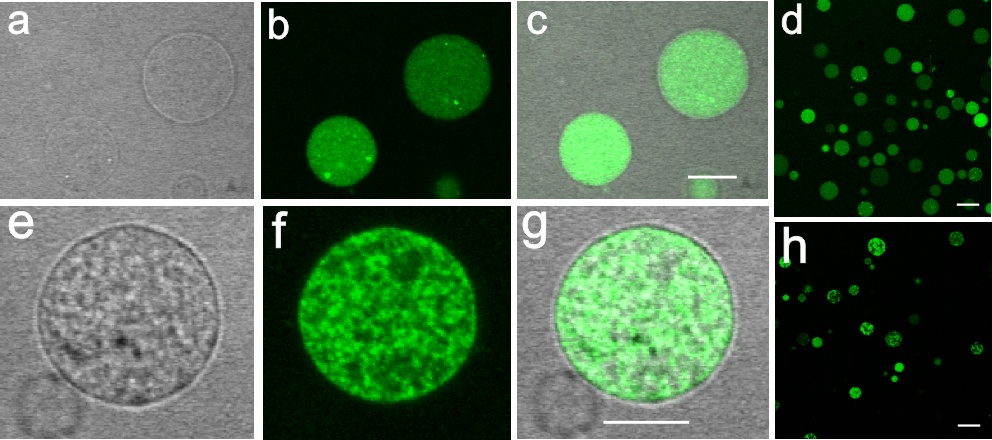
**

**Figure S5.** Confocal images of GMVs encapsulating fluorescein-labeled ssDNA. GMVs incubated with fluorescein-labeled ssDNA for 2 days ((a)-(d)) and 4 days ((e)-(h)) at 37 ^o^C. Scale bar is 10 µm for (a)-(c), (e)-(g) and 20 µm for (d) and (h).


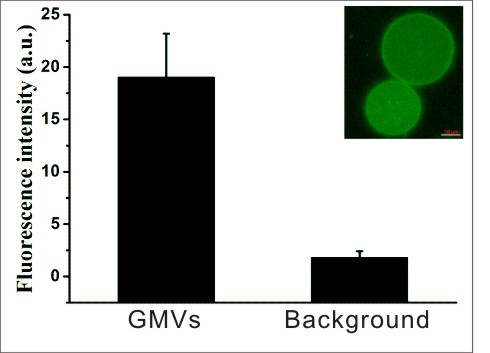


**Figure S6**. Fluorescence intensity of GMVs incubated with 10 µg mL^-1^ curcumin. The packaging of curcumin in GMVs was characterized by calculating the fluorescence intensity inside (GMVs) and outside (Background) of GMVs. More than 80 GMVs were evaluated. Scale bar is 10 µm.

**Table S1.** List of proteins retained in GMVs, as identified by mass spectrometry. More than one thousand proteins were identified and the list below contains the 262 membrane proteins among the total.

| Number | Accession | Description |
| --- | --- | --- |
| 1 | P04406 | Glyceraldehyde-3-phosphate dehydrogenase |
| 2 | P35908 | Keratin, type II cytoskeletal 2 epidermal |
| 3 | P35527 | Keratin, type I cytoskeletal 9 |
| 4 | P08758 | Annexin A5 |
| 5 | P00338 | L-lactate dehydrogenase A chain |
| 6 | P11142 | Heat shock cognate 71 kDa protein |
| 7 | P06733 | Alpha-enolase |
| 8 | P04264 | Keratin, type II cytoskeletal 1 |
| 9 | P46940 | Ras GTPase-activating-like protein IQGAP1 |
| 10 | P07900 | Heat shock protein HSP 90-alpha |
| 11 | P12814-2 | Isoform 2 of Alpha-actinin-1 |
| 12 | P68104 | Elongation factor 1-alpha 1 |
| 13 | P53396-2 | Isoform 2 of ATP-citrate synthase |
| 14 | P27348 | 14-3-3 protein theta |
| 15 | P26641 | Elongation factor 1-gamma |
| 16 | P22234 | Multifunctional protein ADE2 |
| 17 | P04083 | Annexin A1 |
| 18 | P18206-2 | Isoform 1 of Vinculin |
| 19 | P49327 | Fatty acid synthase |
| 20 | P13798 | Acylamino-acid-releasing enzyme |
| 21 | P60842 | Eukaryotic initiation factor 4A-I |
| 22 | P31946-2 | Isoform Short of 14-3-3 protein beta/alpha |
| 23 | Q9UQ80 | Proliferation-associated protein 2G4 |
| 24 | P63244 | Guanine nucleotide-binding protein subunit beta-2-like 1 |
| 25 | P11021 | 78 kDa glucose-regulated protein |
| 26 | P12429 | Annexin A3 |
| 27 | Q00610-2 | Isoform 2 of Clathrin heavy chain 1 |
| 28 | P14625 | Endoplasmin |
| 29 | E9PMI6 | Methylosome subunit pICln |
| 30 | P26038 | Moesin |
| 31 | P23396 | 40S ribosomal protein S3 |
| 32 | Q16555 | Dihydropyrimidinase-related protein 2 |
| 33 | P06744 | Glucose-6-phosphate isomerase |
| 34 | P17655 | Calpain-2 catalytic subunit |
| 35 | P15311 | Ezrin |
| 36 | P13928 | Annexin A8 |
| 37 | P46778 | 60S ribosomal protein L21 |
| 38 | P05186 | Alkaline phosphatase, tissue-nonspecific isozyme |
| 39 | Q99536 | Synaptic vesicle membrane protein VAT-1 homolog |
| 40 | P54819-6 | Isoform 6 of Adenylate kinase 2, mitochondrial |
| 41 | Q92597 | Protein NDRG1 |
| 42 | Q99829 | Copine-1 |
| 43 | Q14232 | Translation initiation factor eIF-2B subunit alpha |
| 44 | Q13200 | 26S proteasome non-ATPase regulatory subunit 2 |
| 45 | Q9Y230 | RuvB-like 2 |
| 46 | P35606-2 | Isoform 2 of Coatomer subunit beta' |
| 47 | P35241 | Radixin |
| 48 | Q9P2E9-3 | Isoform 2 of Ribosome-binding protein 1 |
| 49 | Q15019 | Septin-2 |
| 50 | Q01518 | Adenylyl cyclase-associated protein 1 |
| 51 | P09923 | Intestinal-type alkaline phosphatase |
| 52 | P13647 | Keratin, type II cytoskeletal 5 |
| 53 | Q96QK1 | Vacuolar protein sorting-associated protein 35 |
| 54 | P09104 | Gamma-enolase |
| 55 | Q9Y262-2 | Isoform 2 of Eukaryotic translation initiation factor 3 subunit L |
| 56 | P52597 | Heterogeneous nuclear ribonucleoprotein F |
| 57 | P50395 | Rab GDP dissociation inhibitor beta |
| 58 | P32004-3 | Isoform 3 of Neural cell adhesion molecule L1 |
| 59 | P47755 | F-actin-capping protein subunit alpha-2 |
| 60 | Q02878 | 60S ribosomal protein L6 |
| 61 | P62263 | 40S ribosomal protein S14 |
| 62 | P07237 | Protein disulfide-isomerase |
| 63 | Q9Y490 | Talin-1 |
| 63 | F5H018 | GTP-binding nuclear protein Ran (Fragment) |
| 65 | P33991 | DNA replication licensing factor MCM4 |
| 66 | P33176 | Kinesin-1 heavy chain |
| 67 | Q9ULV4 | Coronin-1C |
| 68 | P31939-2 | Isoform 2 of Bifunctional purine biosynthesis protein PURH |
| 69 | P62847-2 | Isoform 2 of 40S ribosomal protein S24 |
| 70 | P07384 | Calpain-1 catalytic subunit |
| 71 | P11586 | C-1-tetrahydrofolate synthase, cytoplasmic |
| 72 | P12270 | Nucleoprotein TPR |
| 73 | Q00839-2 | Isoform Short of Heterogeneous nuclear ribonucleoprotein U |
| 74 | Q9NVD7-2 | Isoform 2 of Alpha-parvin |
| 75 | O60684 | Importin subunit alpha-7 |
| 76 | P07814 | Bifunctional glutamate/proline--tRNA ligase |
| 77 | P62136-3 | Isoform 3 of Serine/threonine-protein phosphatase PP1-alpha catalytic subunit |
| 78 | Q6UVK1 | Chondroitin sulfate proteoglycan 4 |
| 79 | P05187 | Alkaline phosphatase, placental type |
| 80 | P53621 | Coatomer subunit alpha |
| 81 | P46779-4 | Isoform 4 of 60S ribosomal protein L28 |
| 82 | P60510 | Serine/threonine-protein phosphatase 4 catalytic subunit |
| 83 | P11940-2 | Isoform 2 of Polyadenylate-binding protein 1 |
| 84 | P61160 | Actin-related protein 2 |
| 85 | P36578 | 60S ribosomal protein L4 |
| 86 | P42126-2 | Isoform 2 of Enoyl-CoA delta isomerase 1, mitochondrial |
| 87 | P08195-2 | Isoform 2 of 4F2 cell-surface antigen heavy chain |
| 88 | Q86UP2-2 | Isoform 2 of Kinectin |
| 90 | P17931 | Galectin-3 |
| 91 | P26373 | 60S ribosomal protein L13 |
| 92 | P00505 | Aspartate aminotransferase, mitochondrial |
| 93 | Q6YHK3 | CD109 antigen |
| 94 | Q15233-2 | Isoform 2 of Non-POU domain-containing octamer-binding protein |
| 95 | P01891 | HLA class I histocompatibility antigen, A-68 alpha chain |
| 96 | O15371-2 | Isoform 2 of Eukaryotic translation initiation factor 3 subunit D |
| 97 | P08708 | 40S ribosomal protein S17 |
| 98 | P10809 | 60 kDa heat shock protein, mitochondrial |
| 99 | P30419-2 | Isoform Short of Glycylpeptide N-tetradecanoyltransferase 1 |
| 100 | O43242 | 26S proteasome non-ATPase regulatory subunit 3 |
| 101 | P46379-4 | Isoform 4 of Large proline-rich protein BAG6 |
| 102 | P30040 | Endoplasmic reticulum resident protein 29 |
| 103 | O00299 | Chloride intracellular channel protein 1 |
| 104 | P40763-3 | Isoform 3 of Signal transducer and activator of transcription 3 |
| 105 | P62913-2 | Isoform 2 of 60S ribosomal protein L11 |
| 106 | Q12906-5 | Isoform 5 of Interleukin enhancer-binding factor 3 |
| 107 | Q9BTW9-2 | Isoform 2 of Tubulin-specific chaperone D |
| 108 | Q99460-2 | Isoform 2 of 26S proteasome non-ATPase regulatory subunit 1 |
| 109 | Q9Y678 | Coatomer subunit gamma-1 |
| 110 | P21281 | V-type proton ATPase subunit B, brain isoform |
| 111 | Q96TA1-2 | Isoform 2 of Niban-like protein 1 |
| 112 | O00743-2 | Isoform 2 of Serine/threonine-protein phosphatase 6 catalytic subunit |
| 113 | P35998 | 26S protease regulatory subunit 7 |
| 114 | A8K5M9 | Uncharacterized protein C15orf62, mitochondrial |
| 115 | P52292 | Importin subunit alpha-1 |
| 116 | Q9UNM6 | 26S proteasome non-ATPase regulatory subunit 13 |
| 117 | Q9Y265 | RuvB-like 1 |
| 118 | Q13283 | Ras GTPase-activating protein-binding protein 1 |
| 119 | P62854 | 40S ribosomal protein S26 |
| 120 | Q92973-2 | Isoform 2 of Transportin-1 |
| 121 | Q14204 | Cytoplasmic dynein 1 heavy chain 1 |
| 122 | Q96FM1-2 | Isoform 2 of Post-GPI attachment to proteins factor 3 |
| 123 | Q14152-2 | Isoform 2 of Eukaryotic translation initiation factor 3 subunit A |
| 124 | Q12765-3 | Isoform 3 of Secernin-1 |
| 125 | Q15084-3 | Isoform 3 of Protein disulfide-isomerase A6 |
| 126 | Q15642-5 | Isoform 5 of Cdc42-interacting protein 4 |
| 127 | Q92616 | Translational activator GCN1 |
| 128 | Q9NTK5 | Obg-like ATPase 1 |
| 129 | Q15046 | Lysine--tRNA ligase |
| 130 | O95831 | Apoptosis-inducing factor 1, mitochondrial |
| 131 | P62906 | 60S ribosomal protein L10a |
| 132 | P62195-2 | Isoform 2 of 26S protease regulatory subunit 8 |
| 133 | Q00341 | Vigilin |
| 134 | O60884 | DnaJ homolog subfamily A member 2 |
| 135 | P30740-2 | Isoform 2 of Leukocyte elastase inhibitor |
| 136 | Q10567-3 | Isoform C of AP-1 complex subunit beta-1 |
| 137 | P06737-2 | Isoform 2 of Glycogen phosphorylase, liver form |
| 138 | P49721 | Proteasome subunit beta type-2 |
| 139 | Q6P2Q9 | Pre-mRNA-processing-splicing factor 8 |
| 140 | Q9NRW7-2 | Isoform 2 of Vacuolar protein sorting-associated protein 45 |
| 141 | P13861 | cAMP-dependent protein kinase type II-alpha regulatory subunit |
| 142 | O14579-2 | Isoform 2 of Coatomer subunit epsilon |
| 143 | P11413 | Glucose-6-phosphate 1-dehydrogenase |
| 144 | E9PHH3 | Syndecan-1 |
| 145 | P30050 | 60S ribosomal protein L12 |
| 146 | P10321 | HLA class I histocompatibility antigen, Cw-7 alpha chain |
| 147 | P54136-2 | Isoform Monomeric of Arginine--tRNA ligase, cytoplasmic |
| 148 | P19367-4 | Isoform 4 of Hexokinase-1 |
| 149 | Q9BS26 | Endoplasmic reticulum resident protein 44 |
| 150 | O60784-3 | Isoform 3 of Target of Myb protein 1 |
| 151 | B3KT28 | FAS-associated factor 1 |
| 152 | Q04637-6 | Isoform E of Eukaryotic translation initiation factor 4 gamma 1 |
| 153 | Q13564-3 | Isoform 3 of NEDD8-activating enzyme E1 regulatory subunit |
| 154 | P08754 | Guanine nucleotide-binding protein G(k) subunit alpha |
| 155 | Q08209-3 | Isoform 3 of Serine/threonine-protein phosphatase 2B catalytic subunit alpha isoform |
| 156 | P14061 | Estradiol 17-beta-dehydrogenase 1 |
| 157 | Q02809 | Procollagen-lysine,2-oxoglutarate 5-dioxygenase 1 |
| 158 | P63010-3 | Isoform 3 of AP-2 complex subunit beta |
| 159 | Q9Y295 | Developmentally-regulated GTP-binding protein 1 |
| 160 | Q9NSD9 | Phenylalanine--tRNA ligase beta subunit |
| 161 | E9PLT0 | Cold shock domain-containing protein E1 |
| 162 | P56192 | Methionine--tRNA ligase, cytoplasmic |
| 163 | P17812-2 | Isoform 2 of CTP synthase 1 |
| 164 | Q04917 | 14-3-3 protein eta |
| 165 | P62266 | 40S ribosomal protein S23 |
| 166 | O76094 | Signal recognition particle subunit SRP72 |
| 167 | O00571-2 | Isoform 2 of ATP-dependent RNA helicase DDX3X |
| 168 | P11216 | Glycogen phosphorylase, brain form |
| 169 | Q15075 | Early endosome antigen 1 |
| 170 | Q6P587 | Acylpyruvase FAHD1, mitochondrial |
| 171 | P78527 | DNA-dependent protein kinase catalytic subunit |
| 172 | Q9BXJ9-4 | Isoform 2 of N-alpha-acetyltransferase 15, NatA auxiliary subunit |
| 173 | Q9UBS4 | DnaJ homolog subfamily B member 11 |
| 174 | Q8TD16 | Protein bicaudal D homolog 2 |
| 175 | Q15437 | Protein transport protein Sec23B |
| 176 | P26196 | Probable ATP-dependent RNA helicase DDX6 |
| 177 | P20073-2 | Isoform 2 of Annexin A7 |
| 178 | C9JRH2 | Regulator of chromosome condensation (Fragment) |
| 179 | P17844-2 | Isoform 2 of Probable ATP-dependent RNA helicase DDX5 |
| 180 | P24752 | Acetyl-CoA acetyltransferase, mitochondrial |
| 181 | Q5T4S7-3 | Isoform 3 of E3 ubiquitin-protein ligase UBR4 |
| 182 | P09874 | Poly [ADP-ribose] polymerase 1 (PARP-1) |
| 183 | O14967-2 | Isoform 2 of Calmegin |
| 184 | Q9H8Y8-2 | Isoform 2 of Golgi reassembly-stacking protein 2 |
| 185 | Q9BZJ8 | Probable G-protein coupled receptor 61 |
| 186 | Q8WVM8-2 | Isoform 2 of Sec1 family domain-containing protein 1 |
| 187 | Q96P70 | Importin-9 |
| 188 | Q92945 | Far upstream element-binding protein 2 |
| 189 | O60502-3 | Isoform 3 of Bifunctional protein NCOAT |
| 190 | O00499-9 | Isoform BIN1-10-13 of Myc box-dependent-interacting protein 1 |
| 191 | P13807-2 | Isoform 2 of Glycogen [starch] synthase, muscle |
| 192 | P14868-2 | Isoform 2 of Aspartate--tRNA ligase, cytoplasmic |
| 193 | Q13177 | Serine/threonine-protein kinase PAK 2 |
| 194 | Q14315-2 | Isoform 2 of Filamin-C |
| 195 | Q92878-3 | Isoform 3 of DNA repair protein RAD50 |
| 196 | Q14126 | Desmoglein-2 |
| 197 | P14923 | Junction plakoglobin |
| 198 | P15328 | Folate receptor alpha |
| 199 | P43246-2 | Isoform 2 of DNA mismatch repair protein Msh2 |
| 200 | P46459-2 | Isoform 2 of Vesicle-fusing ATPase |
| 201 | Q6EMK4 | Vasorin |
| 202 | Q9H1Y3 | Opsin-3 |
| 203 | Q08554-2 | Isoform 1B of Desmocollin-1 |
| 204 | Q9UMX0-2 | Isoform 2 of Ubiquilin-1 |
| 205 | Q01650 | Large neutral amino acids transporter small subunit 1 |
| 206 | P40222 | Alpha-taxilin |
| 207 | Q01581 | Hydroxymethylglutaryl-CoA synthase, cytoplasmic |
| 208 | P22059 | Oxysterol-binding protein 1 |
| 209 | O00429-4 | Isoform 3 of Dynamin-1-like protein |
| 210 | O00329 | Phosphatidylinositol 4,5-bisphosphate 3-kinase catalytic subunit delta isoform |
| 211 | Q8TD43-2 | Isoform 2 of Transient receptor potential cation channel subfamily M member 4 |
| 212 | P43681-2 | Isoform 2 of Neuronal acetylcholine receptor subunit alpha-4 |
| 213 | P15924-2 | Isoform DPII of Desmoplakin |
| 214 | O94973-3 | Isoform 3 of AP-2 complex subunit alpha-2 |
| 215 | Q9H3P7 | Golgi resident protein GCP60 |
| 216 | P78347-2 | Isoform 2 of General transcription factor II-I |
| 217 | P32926 | Desmoglein-3 |
| 218 | Q7LFX5-2 | Isoform 2 of Carbohydrate sulfotransferase 15 |
| 219 | Q8N3U4 | Cohesin subunit SA-2 |
| 220 | P51610 | Host cell factor 1 |
| 221 | Q96TA2-3 | Isoform 3 of ATP-dependent zinc metalloprotease YME1L1 |
| 222 | Q86XP3-2 | Isoform 2 of ATP-dependent RNA helicase DDX42 |
| 223 | Q9Y2A7 | Nck-associated protein 1 |
| 224 | O94759-2 | Isoform 2 of Transient receptor potential cation channel subfamily M member 2 |
| 225 | G3V0E5 | Transferrin receptor (P90, CD71), isoform CRA_c |
| 226 | Q02413 | Desmoglein-1 |
| 227 | Q01970-2 | Isoform 2 of 1-phosphatidylinositol 4,5-bisphosphate phosphodiesterase beta-3 |
| 228 | Q66K14-2 | Isoform 2 of TBC1 domain family member 9B |
| 229 | P55265-5 | Isoform 5 of Double-stranded RNA-specific adenosine deaminase |
| 230 | Q08379-2 | Isoform 2 of Golgin subfamily A member |
| 231 | O75054 | Immunoglobulin superfamily member 3 |
| 232 | Q9P265 | Disco-interacting protein 2 homolog B |
| 233 | P42336 | Phosphatidylinositol 4,5-bisphosphate 3-kinase catalytic subunit alpha isoform |
| 234 | Q08378-2 | Isoform 2 of Golgin subfamily A member 3 |
| 235 | Q9Y4D7-2 | Isoform 2 of Plexin-D1 |
| 236 | P47989 | Xanthine dehydrogenase/oxidase |
| 237 | H0Y4H1 | Receptor-type tyrosine-protein phosphatase F (Fragment) |
| 238 | P43121 | Cell surface glycoprotein MUC18 |
| 239 | P06756-3 | Isoform 3 of Integrin alpha-V |
| 240 | P50570-2 | Isoform 2 of Dynamin-2 |
| 241 | Q3MJ16 | Cytosolic phospholipase A2 epsilon |
| 242 | O75436 | Vacuolar protein sorting-associated protein 26A |
| 243 | P19174 | 1-phosphatidylinositol 4,5-bisphosphate phosphodiesterase gamma-1 |
| 244 | Q15323 | Keratin, type I cuticular Ha1 |
| 245 | P61254 | 60S ribosomal protein L26 |
| 246 | P31689-2 | Isoform 2 of DnaJ homolog subfamily A member 1 |
| 247 | Q9NQC3-3 | Isoform 3 of Reticulon-4 |
| 248 | O15355 | Protein phosphatase 1G |
| 249 | Q9Y696 | Chloride intracellular channel protein 4 |
| 250 | P62191-2 | Isoform 2 of 26S protease regulatory subunit 4 |
| 251 | O00232 | 26S proteasome non-ATPase regulatory subunit 12 |
| 252 | P53618 | Coatomer subunit beta |
| 253 | P02792 | Ferritin light chain |
| 254 | Q16658 | Fascin |
| 255 | C9JMZ3 | Aminopeptidase B (Fragment) |
| 256 | P62424 | 60S ribosomal protein L7a |
| 257 | P02538 | Keratin, type II cytoskeletal 6A |
| 258 | P27797 | Calreticulin |
| 259 | P38606-2 | Isoform 2 of V-type proton ATPase catalytic subunit A |
| 260 | B4DUR8 | T-complex protein 1 subunit gamma |
| 261 | P18669 | Phosphoglycerate mutase 1 |
| 262 | P13645 | Keratin, type I cytoskeletal 10 |
